# VO_2_-Based Thermochromic Films Modified by Transparent Matter-Repellent Surfaces with Superior Self-Cleaning Ability and Mechanical Robustness

**DOI:** 10.3390/mi17090997

**Published:** 2026-08-24

**Authors:** Xing Li, Ruizhi Wang, Yukui Cai, Xiaoliang Liang, Yunqing Tang, Jiaqian Li, Zhanqiang Liu

**Affiliations:** 1 School of Mechanical Engineering, Shandong University, Jinan 250061, China; 2 Key Laboratory of High Efficiency and Clean Mechanical Manufacture of Ministry of Education, Jinan 250061, China; 3 School of Nuclear Science, Energy and Power Engineering, Shandong University, Jinan 250061, China

**Keywords:** VO_2_ composite film, VO_2_@SiO_2_, VO_2_@TiO_2_, matter repellence, smart windows

## Abstract

Vanadium dioxide (VO_2_)-based thermochromic films are highly attractive for smart window applications due to their ability to dynamically modulate solar radiation in response to ambient temperature. However, their practical deployment is significantly hindered by poor long-term stability in outdoor environments, vulnerability to surface contamination, and insufficient mechanical durability. Herein, we propose a novel strategy to obtain self-cleaning and durable VO_2_ composite films, consisting of a VO_2_ thermochromic layer covered by either a SiO_2_ or TiO_2_ overcoat and further modified by a transparent and matter-repellent surface. Multifunctional VO_2_ composite films are rationally designed to exhibit superior repellence towards various liquids, thereby imparting excellent anti-fouling property. Crucially, the VO_2_ composite film is engineered for high optical transparency to ensure it does not compromise the solar modulation ability of the underlying VO_2_ layer. Furthermore, the robust mechanical properties of the SiO_2_ or TiO_2_ overcoat with matter-repellent modification provide effective protection against abrasion and scratch damages. The resulting VO_2_-based composite films demonstrate significantly enhanced environmental stability and operational reliability while maintaining desirable thermochromic performance. This work presents a promising strategy to overcome the stability and durability challenges facing VO_2_ smart windows, paving the way for their real-world application in energy-efficient buildings.

## 1. Introduction

With the growing global concern for energy conservation and environmental sustainability, smart windows capable of dynamically modulating solar radiation have become a core component of energy-efficient building envelopes [1,2,3,4,5]. Among various thermochromic materials for smart windows, vanadium dioxide (VO_2_) has attracted extensive attention due to its reversible metal–insulator phase transition at a critical temperature of ~68 °C, which is accompanied by a drastic change in near-infrared (NIR) transmittance [6,7,8,9]. In the semiconductor state (below 68 °C), VO_2_ films allow NIR light to pass through, facilitating indoor heating in cold environments; in the metallic state (above 68 °C), they block NIR light, suppressing excessive indoor heating in hot weather, thus achieving energy-saving effects without additional energy input [10].

Nevertheless, the large-scale commercialization of pristine VO_2_ thermochromic films is still severely limited by three inherent drawbacks: poor long-term environmental stability (susceptible to oxidation and degradation under ambient humidity and UV radiation); vulnerability to surface contamination by dust, water, and oily liquids; and insufficient mechanical robustness against abrasion and scratches in outdoor service. These issues collectively degrade the service performance and lifespan of VO_2_-based smart windows [11].

Surface modification has been recognized as an effective approach to tackle the above challenges [12,13,14,15]. Among various surface modification approaches, the construction of protective layers and transparent matter-repellent surfaces have gained increasing interest [16,17]. Protective layers, including SiO_2_, TiO_2_, AlN, and their composites, can prevent VO_2_ films from oxidizing and degrading in the environmental atmosphere [18,19,20,21,22,23,24,25,26]. Transparent matter-repellent surfaces can endow these films with self-cleaning, anti-contamination, and anti-corrosion capabilities while maintaining their intrinsic thermochromic properties [27,28,29,30].

Despite these advances, most existing matter-repellent modified VO_2_ films suffer from a trade-off between transparency, matter repellence, and mechanical robustness; some modified films exhibit excellent repellency but poor optical transparency due to the rough surface structure, while others have high transparency but weak mechanical stability, leading to the easy damage of the repellent layer. Additionally, the thermodynamical instability of VO_2_ itself, which is prone to oxidation in the ambient air, further exacerbates the degradation of film performance over time, making the development of a transparent and protective overcoat with both superior matter repellence and mechanical robustness an urgent need for the practical application of VO_2_-based smart windows.

Hence, this work proposes a novel triple-layer architecture strategy for VO_2_ thermochromic composite films, aiming to integrate excellent thermochromic performance, superior matter repellence, and high mechanical robustness into VO_2_-based films. The transparent matter-repellent top layer is designed to not only repel various contaminant with a high contact angle and low sliding angle but also maintain high visible light transmittance, ensuring that the intrinsic optical switching performance of VO_2_ is not compromised. The transparent SiO_2_ or TiO_2_ middle layer is designed to enhance the thermochromic properties of the underlying VO_2_ layer. Moreover, the top and middle modification layers are engineered to possess enhanced mechanical robustness, enabling them to resist mechanical scratches, water impinging, and environmental erosion, thus extending the service life of the VO_2_ thermochromic films. This study is expected to provide a feasible and effective strategy for overcoming the degradation problem of VO_2_-based thermochromic films, promote their practical application in smart windows, and offer new insights into the surface modification of functional films for energy-saving building materials.

## 2. Experimental Methods

### 2.1. Materials

Tetraethyl orthosilicate, anhydrous ethanol, oxalic acid dihydrate, acetylacetone, dimethyldimethoxysilane, ammonia water, and methyl silicone oil with a viscosity of 20 cSt at 25 °C were purchased from Shanghai Merrell Chemicals Co., Ltd. (Shanghai, China). Vanadium pentoxide was purchased from Anhui Zesheng Technology Co., Ltd. (Anqing, China). Quartz glass was purchased from Wuxi Aurui Tuo Analytical Instrument Technology Co., Ltd. (Wuxi, China). Sulfuric acid was purchased from Jinan Qiguang Technology and Trade Co., Ltd. (Jinan, China). All reagents used in this study were of analytical grade with a purity of 99.7%.

### 2.2. Preparation of Matter-Repellent VO_2_@SiO_2_ and VO_2_@TiO_2_ Composite Films

#### 2.2.1. Fabrication of VO_2_ Underlying Layer

Firstly, 6.3 g of oxalic acid dihydrate and 4.6 g of vanadium pentoxide were placed into a round-bottom flask, followed by the addition of 100 mL of anhydrous ethanol. Then, the obtained mixture was stirred and refluxed for 12 h in a constant temperature oil bath set at 100 °C. After allowing the mixture to settle, the supernatant was collected as the precursor sol. After that, a quartz glass substrate was cleaned in a plasma cleaner at a power of 25 W for 3 min. It was then placed on a benchtop spin coater (KW-4A, Institute of Microelectronics, Chinese Academy of Sciences, Beijing, China), secured under vacuum, and spun at 2000 r/s. Upon reaching the set speed, 0.2 mL of the precursor sol was dropped onto the center of the quartz glass using a pipette. Finally, the spin-coated sample was subsequently placed in a five-temperature zone tube furnace (OTF-1200X-V, Institute of Microelectronics, Chinese Academy of Sciences, China) and purged with air at a flow rate of 5 sccm. The temperature in the furnace was programmed in three stages: the first stage involved heating from 0 to 150 °C for 15 min; the second stage involved heating from 150 °C to 450 °C for 150 min; and the third stage maintained a constant temperature of 450 °C for 120 min. After the process was completed, the sample was allowed to cool naturally in the furnace and the VO_2_ underlying layer was thus obtained.

#### 2.2.2. Fabrication of VO_2_@SiO_2_ and VO_2_@TiO_2_ Composite Films

In order to increase the stability of the VO_2_ thermochromic layer, SiO_2_ and TiO_2_ overcoats were designed to cover the VO_2_ layer. Firstly, SiO_2_ precursor sol and TiO_2_ precursor sol were prepared. Tetraethyl orthosilicate, anhydrous ethanol, ammonia solution, and deionized water were added to a beaker at a volume ratio of 1:10:0.3:0.1 and stirred until homogeneous. After allowing the mixture to settle, the supernatant was collected as the SiO_2_ precursor sol. Titanium (IV) butoxide, anhydrous ethanol, acetylacetone, and deionized water were added to a beaker at a volume ratio of 1:10:0.3:0.1 and stirred until homogeneous. After allowing the mixture to settle, the supernatant was collected as the TiO_2_ precursor sol. Then, the substrates with the VO_2_ layers were placed on the spin coater and secured under vacuum. After that, 0.2 mL of the SiO_2_ or TiO_2_ precursor sol was dropped onto the center of the corresponding substrates with a pipette. Finally, the substrates were transferred to an oven and dried at 100 °C for 30 min, thereby obtaining the VO_2_@SiO_2_ and VO_2_@TiO_2_ composite films.

#### 2.2.3. Matter-Repellent Modification of VO_2_@SiO_2_ and VO_2_@TiO_2_ Surfaces

For the sake of endowing VO_2_@SiO_2_ and VO_2_@TiO_2_ composite films with self-cleaning and wear-resistance abilities, matter-repellent surfaces were designed to encapsulate the VO_2_@SiO_2_ and VO_2_@TiO_2_ composite films. Firstly, dimethoxydimethylsilane and concentrated sulfuric acid were mixed at a volume ratio of 5:1 and shaken thoroughly until a stable silane solution was formed. Then, VO_2_@SiO_2_ and VO_2_@TiO_2_ were rinsed sequentially with deionized water and anhydrous ethanol, followed by drying with blow air. After that, the prepared silane solution was dropped onto the surfaces of VO_2_@SiO_2_ and VO_2_@TiO_2_ composite films to ensure thorough contact, and the samples were allowed to stand for 3 min to facilitate complete reaction between the solution and the surfaces. Finally, the obtained samples were rinsed sequentially with distilled water and anhydrous ethanol, and then dried. Once the substrate was dry, silicone oil was sprayed onto the surface of the substrates, thereby obtaining the matter-repellent VO_2_@SiO_2_ and VO_2_@TiO_2_ films.

### 2.3. Characterizations of VO_2_-Based Composite Films

A tungsten filament scanning electron microscope (SEM; JSM-7610F, Japan Electron Optics Laboratory Co., Ltd., Tokyo, Japan) and energy dispersive spectrometer (EDS) were employed to observe the surface morphologies and elemental compositions of the samples. EDS analysis was performed on three random areas of each surface to identify the constituent elements and their respective contents. Transmission electron microscopy (TEM; Talos F200S G2, Thermo Fisher Scientific, Waltham, MA, USA) was employed to further observe the morphologies of the samples, and selected area electron diffraction (SAED) was performed at an accelerating voltage of 200 kV. Laser scanning confocal microscopy (LSCM; VK-X260, KEYENCE, Osaka, Japan) was utilized to measure the surface roughness of the samples. Each test was repeated three times to calculate the mean value and standard error of surface roughness.

The phase compositions of VO_2_-based composite films were further investigated by X-ray diffraction (XRD; D8 Advance, Bruker, Billerica, MA, USA) using Cu Kα radiation. The XRD measurements were carried out at an operating voltage of 40 kV and a current of 40 mA, with a scanning range of 2θ from 10° to 80° and a scanning speed of 1°/min. The surface chemical structures of the samples were characterized by Fourier transform infrared spectroscopy (FTIR, Nicolet iS20, Thermo Fisher Scientific, USA). The contact angle measurements were conducted using a contact angle goniometer (SZ-CAMC33, Shanghai Xuanzhun Instrument Co., Ltd., Shanghai, China). A 6 μL droplet of deionized water was placed on the sample surface, and the contact angle was measured at five randomly selected points to achieve a mean value and a standard error for each sample.

In order to evaluate the optical performance of VO_2_-based composite films, the visible light transmittance (T_lum_, 380 to 780 nm) and solar transmittance (T_sol_, 260 to 2600 nm) of the VO_2_-based composite films were measured at different temperatures using a spectrophotometer (Lambda 950, PerkinElmer, Norwalk, CT, USA). The haze and scattering rate of the films were characterized and statistically calculated. Firstly, the incident light flux (T_1_) was measured with no sample placed under the light trap with the port closed. Then, keeping the same optical path configuration but placing the sample in the transmission chamber, the total transmitted light flux (T_3_) passing through the sample was measured. Next, with the sample removed and the light trap port open, the scattering light flux (T_2_) of the instrument itself was measured. Finally, the sample was placed with the light trap port open, and the scattering light flux (T_4_) produced by the instrument and the sample was measured. Based on the above results, the haze value (H) could be calculated by the following formula.

(1) $$H=(\frac{T_{4}}{T_{2}}-\frac{T_{3}}{T_{1}})\times 100$$

Since T_lum_ and solar modulation ability (ΔT_sol_) are critical factors for evaluating the ability to regulate solar heat, we calculated T_lum_ and ΔT_sol_ based on their ultraviolet–visible–near-infrared (UV-Vis-NIR) transmittance spectra. The formulas for calculating these are as follows:

(2) $$T_{\mathrm{lum},\mathrm{sol}\;}=\int \varphi_{\mathrm{lum},\mathrm{sol}\;}(\lambda )T(\lambda )d\lambda /\int \varphi_{\mathrm{lum},\mathrm{sol}\;}(\lambda )d\lambda$$

(3) $$\Delta T_{\mathrm{sol}\;}=T_{\mathrm{sol}\;}\left(25\;{}^{\circ}C\right)-T_{\mathrm{sol}\;}\left(95\;{}^{\circ}C\right)$$

where λ is wavelength, T(λ) denotes the transmittance at λ, φ_lum_(λ) represents the photopic luminous efficiency function of the human eye, and φ_sol_(λ) represents the solar irradiance spectrum at an air mass of 1.5 corresponding to a sun position of 37° above the horizon. Standard errors of haze values were obtained from the mean of three replicate measurements.

## 3. Results and Discussion

### 3.1. Surface Morphologies and Chemical Compositions of VO_2_-Based Composite Films

**Figure 1:**
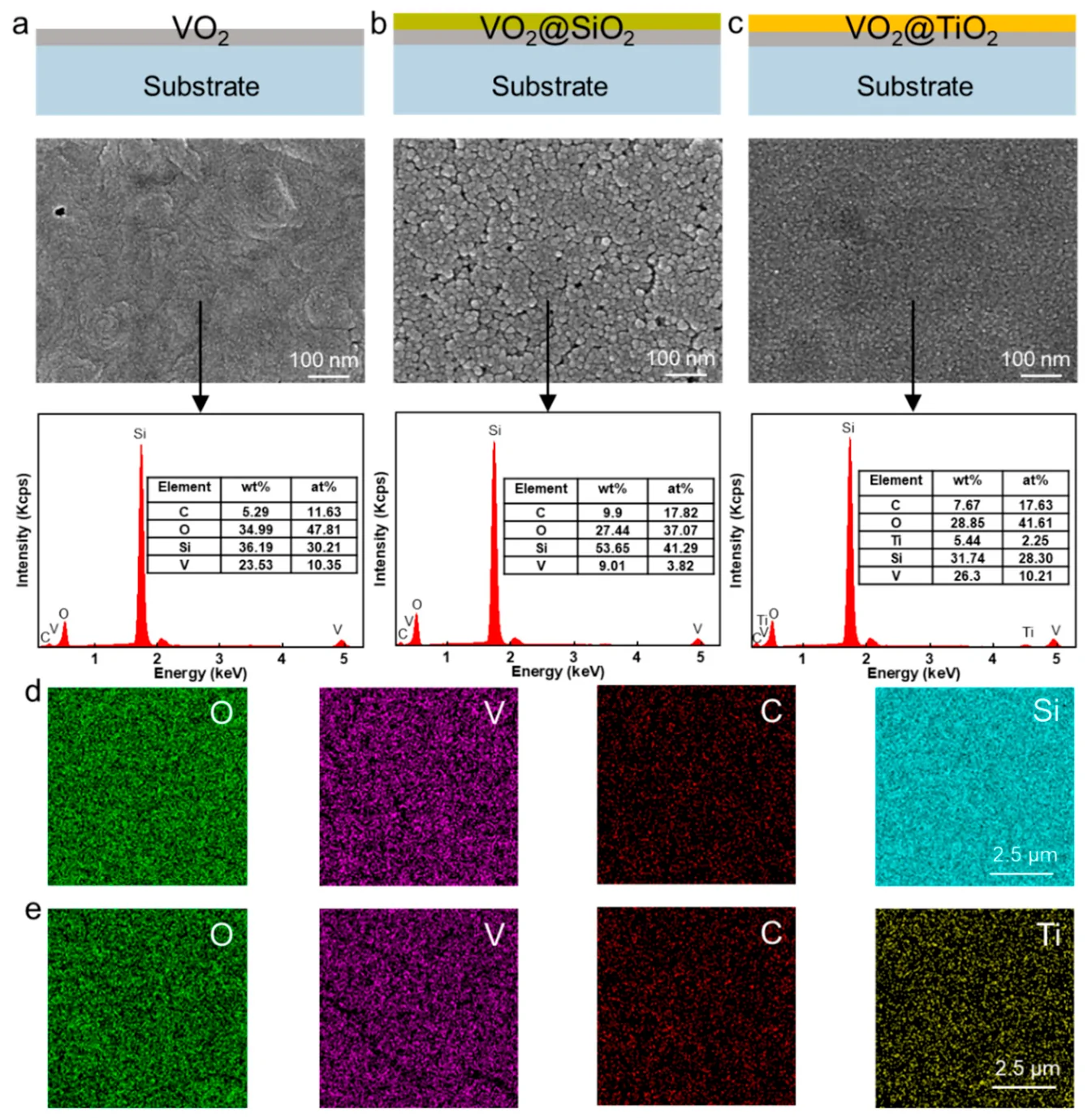
SEM images and corresponding EDS statistical results of elemental content of the (**a**) VO_2_, (**b**) VO_2_@SiO_2_, and (**c**) VO_2_@TiO_2_ films; EDS mapping images of (**d**) the VO_2_@SiO_2_ film and (**e**) the VO_2_@TiO_2_ film.

To develop matter-repellent VO_2_-based composite films, we start by depositing a SiO_2_ layer or a TiO_2_ layer upon the VO_2_ underlying layer, thus obtaining VO_2_@SiO_2_ films and VO_2_@TiO_2_ films. Figure 1 shows the scanning electron micrograph and EDS results of the VO_2_, VO_2_@SiO_2_, and VO_2_@TiO_2_ films. For each SEM sample, three different regions were selected for measurement, and the elemental contents showed little difference among these regions. Figure 1a presents an SEM image of the VO_2_ thin film, revealing that nano-scale VO_2_ particles uniformly distribute across the entire substrate surface. Figure 1b,c show SEM images of the VO_2_@SiO_2_ and VO_2_@TiO_2_ films, respectively, indicating that the VO_2_ particles are encapsulated by transparent SiO_2_ and TiO_2_ nanoparticles. These encapsulated SiO_2_ and TiO_2_ particles exhibit an irregular spherical morphology without significant aggregation. The particle size distribution is relatively homogeneous, with diameters significantly below 40 nm. According to the theory of Laaksonen on the transmittance of VO_2_ coatings, it should be acknowledged that when the particle size is less than 40 nm, the transmittance of the coating at shorter wavelengths remains unaffected. Figure 1d,e display elemental mapping images of the VO_2_@SiO_2_ and VO_2_@TiO_2_ films, respectively, demonstrating that the primary elements of the prepared VO_2_, VO_2_@SiO_2_, and VO_2_@TiO_2_ films are uniformly distributed across the substrate surface with no element enrichment or depletion zones.

**Figure 2:**
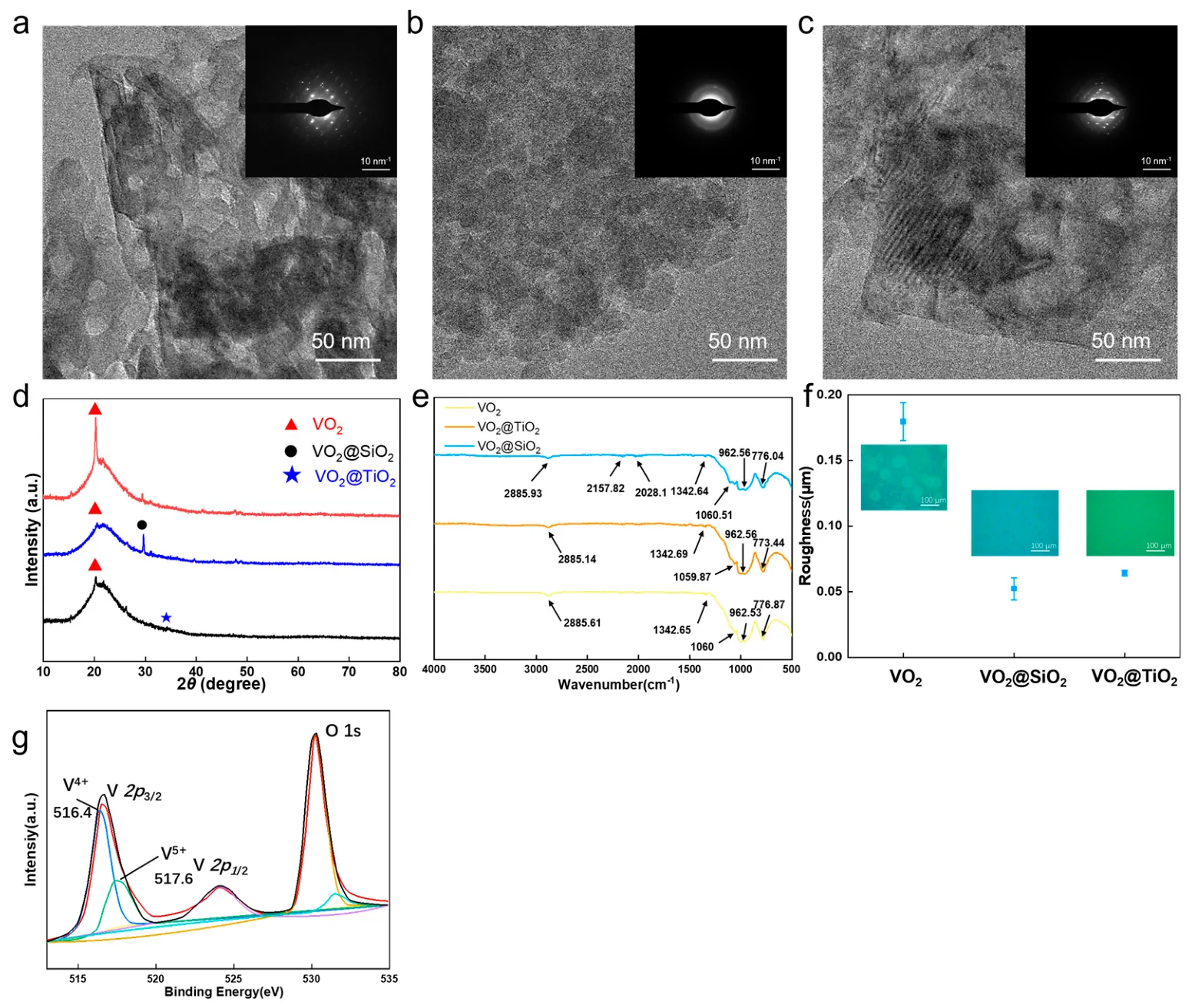
TEM and SAED images of the (**a**) VO_2_, (**b**) VO_2_@SiO_2_, and (**c**) VO_2_@TiO_2_ films; and the (**d**) XRD spectra, (**e**) FTIR spectra, and (**f**) surface roughness of the VO_2_, VO_2_@SiO_2_, and VO_2_@TiO_2_ films and (**g**) XPS spectra of VO_2_.

To further determine the structures of obtained VO_2_-based films, TEM scanning, XRD, FTIR, and surface roughness experiments were performed on the samples, and the results are shown in Figure 2. For each type of sample, three independent measurements were conducted for both XRD and FTIR, and the positions of the characteristic peaks were found to be essentially identical across the three tests. The TEM image of the VO_2_ film, as shown in Figure 2a, reveals its nanoporous structure. This nanoporous structure significantly increases the specific surface area of the VO_2_ film, facilitating rapid heat exchange with the external environment and enhancing the response speed of the phase transition. Figure 2b presents the TEM image of the VO_2_@SiO_2_ film, showing SiO_2_ particles covering the surface in granular form. The corresponding diffraction pattern confirms that the SiO_2_ particles are amorphous. The TEM image of the VO_2_@TiO_2_ film, displayed in Figure 2c, reveals a striped structure of TiO_2_, through which the porous morphology of the underlying VO_2_ film can be observed. The corresponding diffraction pattern indicates the presence of two crystalline phases, confirming the successful preparation of the VO_2_@TiO_2_ composite film.

In order to further explore the chemical compositions of VO_2_-based films, XRD and FTIR analysis were conducted, and the results are presented in Figure 2d,e. The XRD patterns of the VO_2_, VO_2_@SiO_2_, and VO_2_@TiO_2_ films indicate that the VO_2_ structure remains unchanged and retains the M phase with the SiO_2_ and TiO_2_ overcoats. In addition, a diffraction peak appearing at 30.16° corresponds to SiO_2_ of the VO_2_@SiO_2_ film, while a peak at 34.87° is attributed to TiO_2_ of the VO_2_@TiO_2_ film [31]. The functional groups of VO_2_-based films are revealed by FTIR, and the results are shown in Figure 2d. These three samples exhibit prominent absorption peaks at 1343 cm^−1^ and 2885 cm^−1^, corresponding to the C–O and C–H bonds, respectively, which originate from residual organic solvents that were not fully decomposed during film preparation. In the VO_2_ film, the absorption peak at 776.87 cm^−1^ is attributed to the symmetric vibration of Si–O–Si, while the peak at 1060 cm^−1^ corresponds to the asymmetric vibration of Si–O–Si, both originating from the quartz glass substrate. The absorption peak at 962.53 cm^−1^ is associated with the V=O bond vibration, confirming the formation of the VO_2_ lattice. In the VO_2_@SiO_2_ film, the intensities of the Si–O–Si absorption peaks at 776.04 cm^−1^ and 1060.51 cm^−1^ are significantly enhanced due to the formation of SiO_2_, which amplifies the Si–O–Si signal [32]. In the VO_2_@TiO_2_ film, the absorption peak at 773.44 cm^−1^ corresponds to the Ti–O–Ti symmetric vibration, confirming the formation of the TiO_2_ structure. The surface roughness of the samples is further measured, as shown in Figure 2f. The VO_2_ film exhibits a relatively rough surface. After coating with SiO_2_ and TiO_2_ particles, the roughness of the films is significantly reduced, resulting in a smoother surface of the VO_2_@SiO_2_ and VO_2_@TiO_2_ films. The samples were characterized by XPS, and the results are shown in Figure 2g. It can be seen that the V 2p_3/2_ peak can be deconvoluted into two peaks at binding energies of 516.4 eV for V^4+^ and 517.6 eV for V^5+^. V^4+^ corresponds to the as-prepared VO_2_ thin film, while V^5+^ arises from oxidation to V_2_O_5_ during annealing or upon storage in air.

### 3.2. Thermally Induced Phase Transformation Performance for Smart Windows

**Figure 3:**
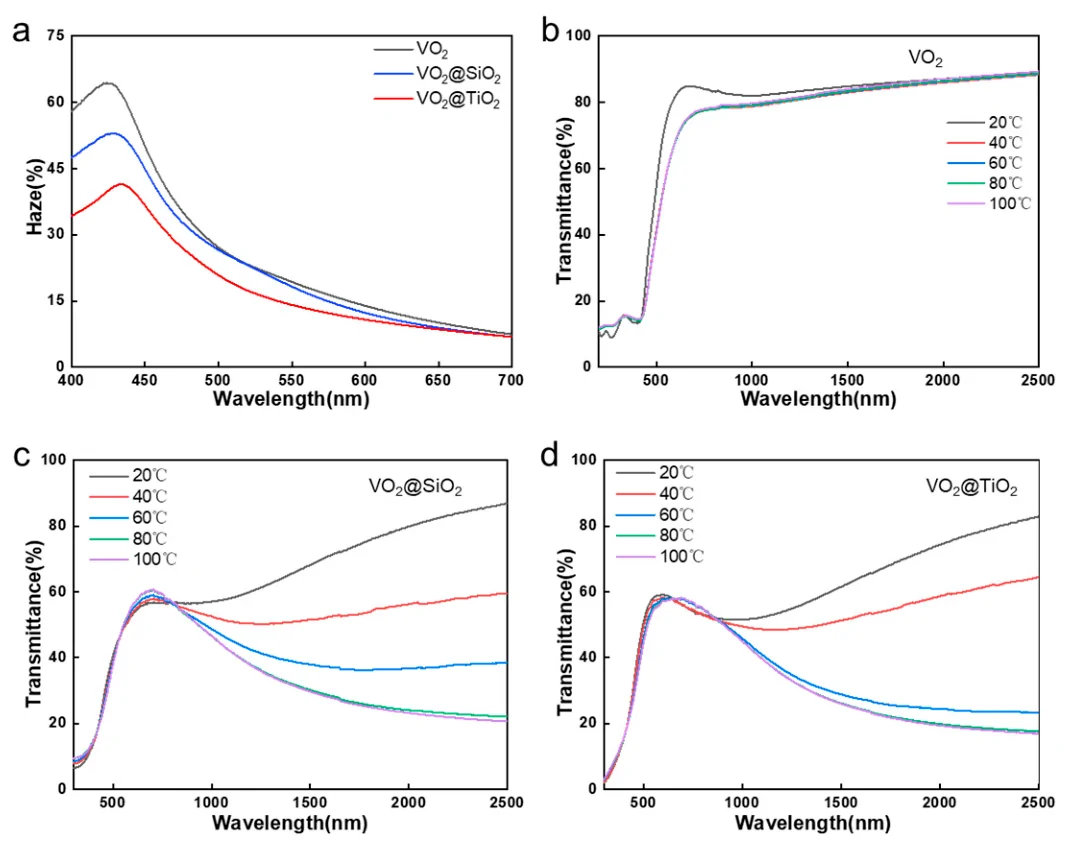
(**a**) Haze of the VO_2_, VO_2_@SiO_2_, and VO_2_@TiO_2_ films; transmittance under different temperatures of the (**b**) VO_2_ film (**c**) VO_2_@SiO_2_ film, and (**d**) VO_2_@TiO_2_ film.

As an exterior building envelope component, the primary function of smart windows is to regulate heat while ensuring clear and comfortable daylighting and views, which necessitates low haze. Figure 3a presents the results of haze tests and analyses, showing that the VO_2_@SiO_2_ and VO_2_@TiO_2_ films exhibit reduced haze values by 1.1% and 3.9%, respectively, compared to that of the VO_2_ film. For each type of sample, three different regions were selected for haze and transmittance measurements, and the results were essentially identical. Subsequently, the transmittances of the samples were investigated, and the results are shown in Figure 3b–d. For the calculation of optical properties, three independent samples of each type were tested, and the reported data are the average values. It is noted that, for Figure 3, deviations of the individual transmittance and haze curves from their averages were within 5%.

The optical properties of the films were then calculated based on Equations (2) and (3) and are listed in Table 1. And the standard errors of T_lum_ and T_sol_ are obtained from the mean of three replicated tests. As shown in Table 1, ΔT_sol_ of the VO_2_@SiO_2_ and VO_2_@TiO_2_ composite films increased by 84% and 72%, respectively, compared to that of the VO_2_ film, indicating that the covered SiO_2_ or TiO_2_ layer significantly enhanced the core temperature control performance of the VO_2_ film. Furthermore, T_lum_ of the VO_2_ film decreased from 73.5% at 25 °C to 58.4% at 95 °C, representing a reduction of 20.5%. After the SiO_2_ and TiO_2_ coatings were prepared, they acted as a refractive-index buffer layer between the VO_2_ and air, establishing a continuous refractive-index gradient. At low temperatures, the refractive indices of the SiO_2_ and TiO_2_ coatings lay between those of air and VO_2_, reducing the index mismatch and producing a destructive interference of reflected light. At high temperatures, VO_2_ transformed into the metallic phase, greatly enhancing its intrinsic near-infrared absorption; combined with the improved reflective performance of the multilayer stack, the overall effect is further strengthened. In both cases, the transmittance difference was significantly widened, directly boosting ΔT_sol_. In contrast, T_lum_ of the VO_2_@SiO_2_ film showed no significant attenuation and even slightly improved, while that of the VO_2_@TiO_2_ film decreased by only 3.3%. These results suggest that the SiO_2_ and TiO_2_ coating layers effectively mitigated the degradation of optical transmittance in VO_2_ films at high temperatures, enabling the VO_2_@SiO_2_ and VO_2_@TiO_2_ composite films to achieve both efficient regulation and excellent visual transparency in smart temperature control applications.

### 3.3. Self-Cleaning and Mechanical Robustness

**Figure 4:**
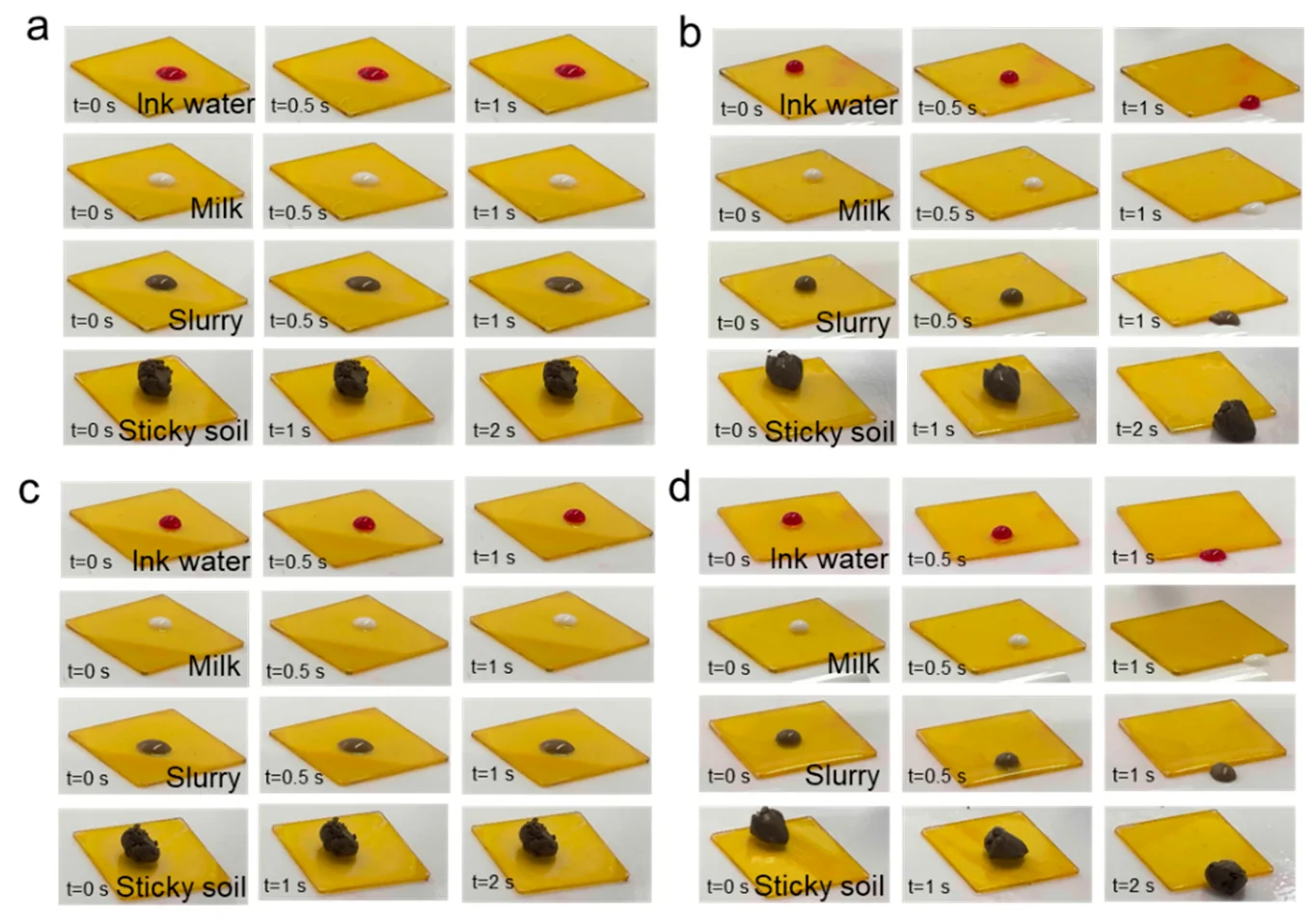
Self-cleaning abilities of the (**a**) VO_2_@SiO_2_ film, (**b**) M-VO_2_@SiO_2_ film, (**c**) VO_2_@TiO_2_ film, and (**d**) M-VO_2_@TiO_2_ film.

For the sake of endowing VO_2_-based composite films with superior self-cleaning properties and mechanical robustness, matter-repellent surface treatments were conducted on VO_2_@SiO_2_ and VO_2_@TiO_2_ films, thus creating the M-VO_2_@SiO_2_ and M-VO_2_@TiO_2_ films. As shown in Figure 4a,c, ink water droplet, milk droplet, slurry droplet, and sticky soil were adhered to the VO_2_@SiO_2_ and VO_2_@TiO_2_ films. In comparison, as shown in Figure 4b,d, the droplets and sticky soil slid off the surfaces of VO_2_@SiO_2_ and VO_2_@TiO_2_ films without leaving residues after matter-repellent treatments.

**Figure 5:**
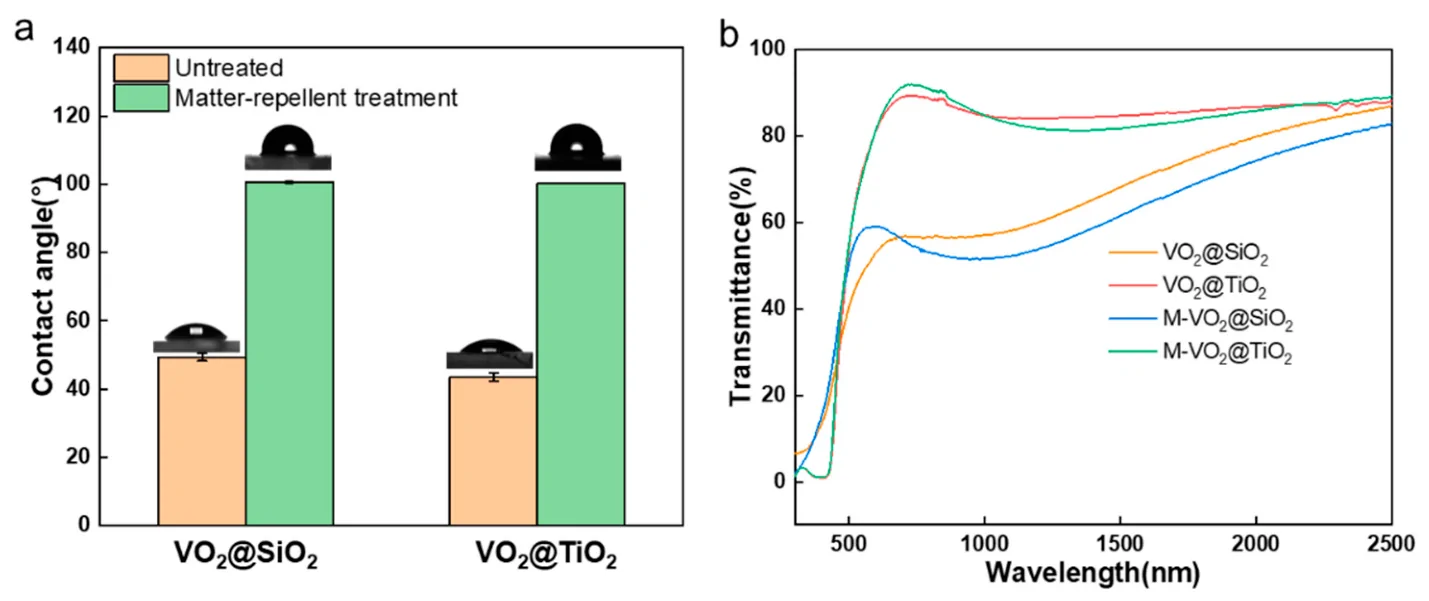
(**a**) Contact angles and (**b**) transmittances of the VO_2_@SiO_2_, M-VO_2_@SiO_2_, VO_2_@TiO_2_, and M-VO_2_@TiO_2_ films.

The contact angles of the composite films before and after matter-repellent treatments were also measured, and the results are shown in Figure 5a. The contact angles of VO_2_@SiO_2_ and VO_2_@TiO_2_ films change from 49.3° and 43.6° to 100.6° and 100.2°of the M-VO_2_@SiO_2_ and M-VO_2_@TiO_2_ films, respectively, transitioning from hydrophilic surfaces to hydrophobic surfaces. Subsequently, the transmittances of the M-VO_2_@SiO_2_ and M-VO_2_@TiO_2_ films were tested and compared with the VO_2_@SiO_2_ and VO_2_@TiO_2_ films, as shown in Figure 5b. For transmittance measurements, three independent samples of each type were tested, and the deviations of the individual transmittance curves from their averages were within 5%. The transmittance of the VO_2_@SiO_2_ film increased in the visible light range after matter-repellent treatment, while the transmittance in the non-visible light range decreased by no more than 5%. The transmittance of VO_2_@TiO_2_ film remained similar before and after super-slippery treatment, indicating that the matter-repellent treatment had little impact on the transmittance of VO_2_-based films.

**Figure 6:**
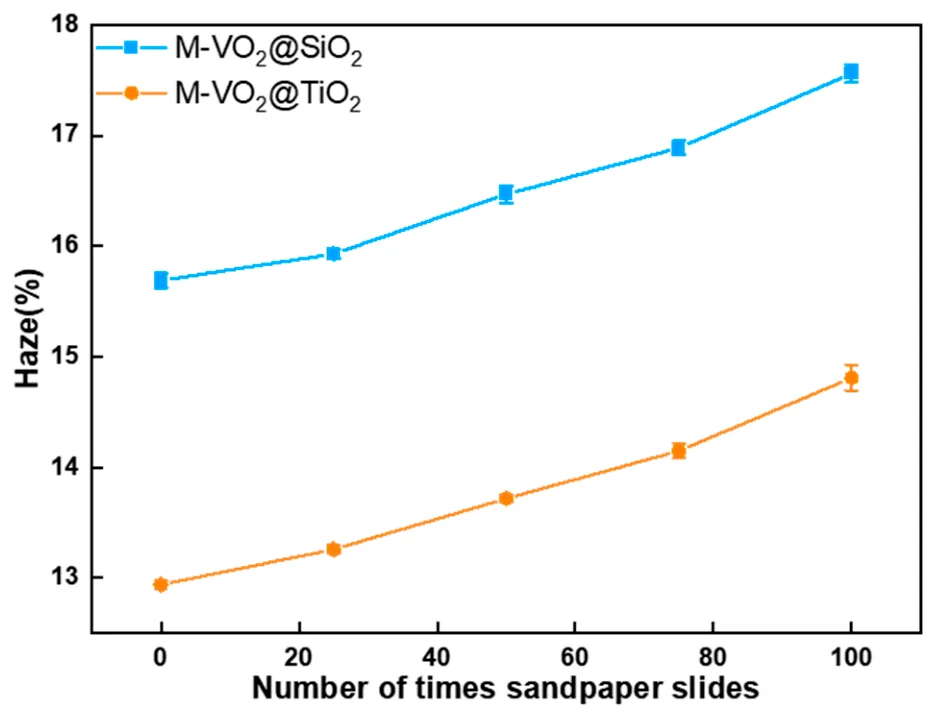
Hazes of M-VO_2_@SiO_2_ and M-VO_2_@TiO_2_ films under different numbers of friction cycles.

To characterize the mechanical durability of the M-VO_2_@SiO_2_ and M-VO_2_@TiO_2_ films, sandpaper abrasion experiments were conducted. Briefly, 2000# sandpaper was placed above the sample, and then a force of 3 N was applied above the sandpaper. Afterwards, the sample was dragged forward at a speed of 0.03 m/s until it was completely pulled away. As shown in Figure 6, after the sample underwent 100 cycles of friction, the haze loss was less than 2%, which indicated good mechanical robustness of the M-VO_2_@SiO_2_ and M-VO_2_@TiO_2_ films.

**Figure 7:**
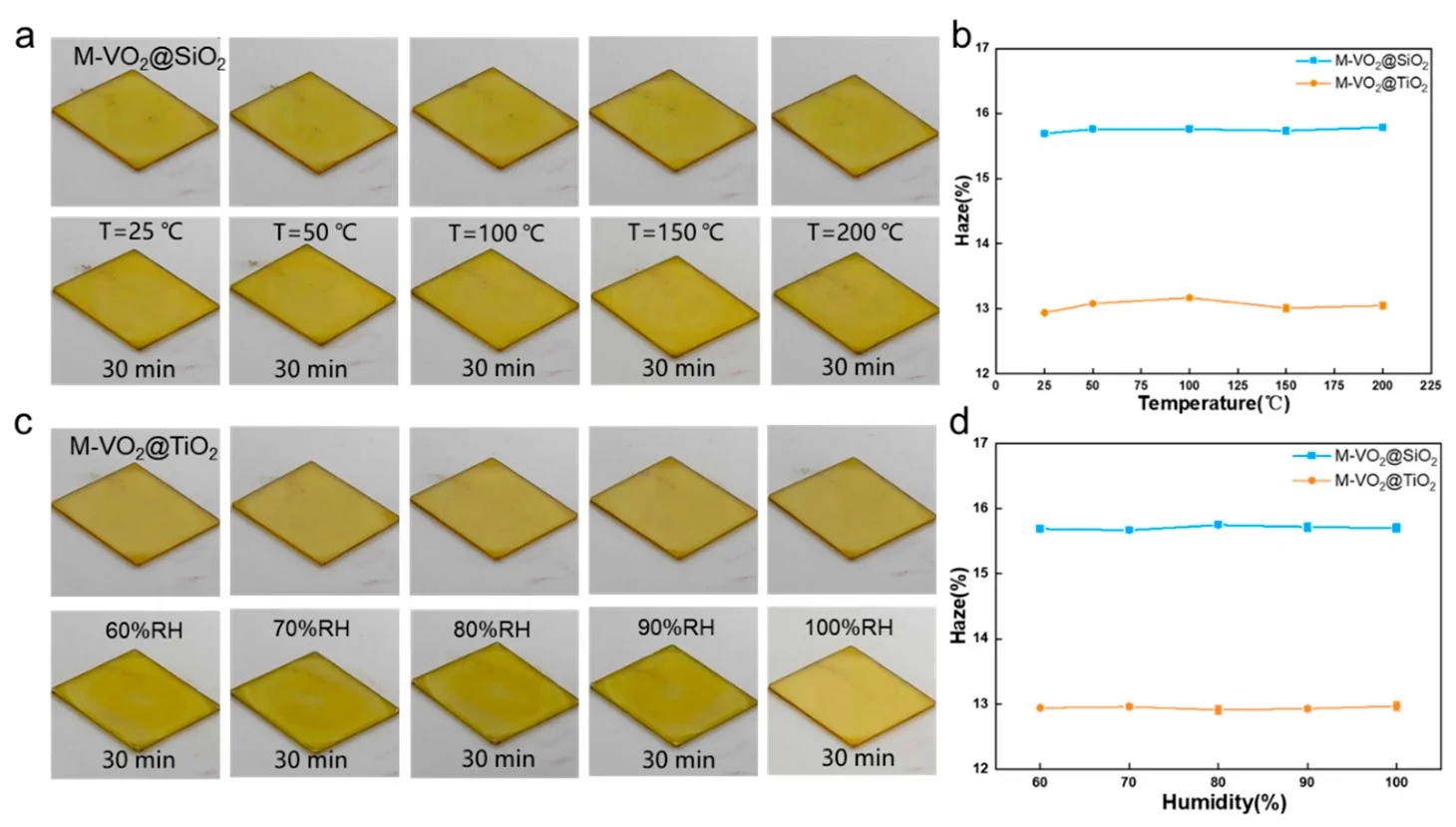
(**a**) Appearance of M-VO_2_@SiO_2_ under different temperatures, (**b**) hazes of M-VO_2_@SiO_2_ and M-VO_2_@TiO_2_ under different temperatures, (**c**) appearance of M-VO_2_@TiO_2_ under different humidities, and (**d**) hazes of M-VO_2_@SiO_2_ and M-VO_2_@TiO_2_ under different humidities.

The physical structural stability of VO_2_-based films under actual service conditions is also crucial for their reliable operation. The damage caused by damp, heat, and high temperature stress to VO_2_-based functional films directly leads to a series of visible physical morphological degradation and haze changes. As shown in Figure 7a,c, after being placed under different temperatures and humidities for thirty minutes, the M-VO_2_@SiO_2_ and M-VO_2_@TiO_2_ films exhibit no macroscopic physical degradation—specifically, no color change, no bubble formation, no turbidity or fogging, and no coating peeling or cracking. In addition, it can be summarized from Figure 7b,d that the haze values of the M-VO_2_@SiO_2_ and M-VO_2_@TiO_2_ films barely change after being placed at different temperatures and humidities for 30 min. No physical morphological degradation and litter haze changes indicate excellent stability of the M-VO_2_@SiO_2_ and M-VO_2_@TiO_2_ films under the effects of damp and heat. In addition, the M-VO_2_@SiO_2_ and M-VO_2_@TiO_2_ thin films were subjected to ultraviolet irradiation for 140 h at 85% relative humidity. Ultraviolet irradiation was achieved by adopting a violet light lamp with a power of 20 W, a wavelength of 395 nm, and an irradiation distance of 2 cm. Neither evident morphological variation nor significant shift in haze values was detected for the samples prior to and after the aging treatment.

## 4. Conclusions

In this study, we have developed an effective strategy for obtaining VO_2_-based thermochromic films with a superior self-cleaning ability and mechanical robustness. The thermochromic VO_2_ underlying layer is shielded by SiO_2_ and TiO_2_ transparent nanoparticles and further subjected to matter-repellent surface treatments. Physical and chemical characterizations demonstrate that these encapsulated SiO_2_ and TiO_2_ particles exhibit an irregular spherical morphology without significant aggregation, with homogeneous particle size distributions and diameters significantly below 40 nm. In comparison with the pure VO_2_ film, the ΔT_sol_ of the VO_2_@SiO_2_ and VO_2_@TiO_2_ composite films increased by 84% and 72%, respectively, indicating the improvement of the core temperature control performance. The contact angle and transmittance experimental results prove that further matter-repellent surface treatment endows VO_2_@SiO_2_ and VO_2_@TiO_2_ films with superior self-cleaning properties in keeping with their excellent thermochromic abilities. The friction and environment experimental results demonstrate that after matter-repellent surface treatment, the VO_2_@SiO_2_ and VO_2_@TiO_2_ films retain superior mechanical robustness, which makes VO_2_@SiO_2_ and VO_2_@TiO_2_ films able to undergo 100 cycles of friction and remain stable under the effects of damp and heat. This study provides an effective method to produce VO_2_-based composite films with superior self-cleaning abilities and mechanical robustness and proves their potential as thermochromic materials, which is expected to broaden the application of VO_2_-based composite films in the smart window area.

## Figures and Tables

**Table 1:** Optical parameters of VO_2_, VO_2_@SiO_2_, and VO_2_@TiO_2_ films.

Sample	*T*_lum_ (%)		*T*_sol_ (%)		Δ*T*_sol_ (%)
	25 °C	95 °C	25 °C	95 °C	
VO_2_	73.5 ± 1.5	58.4 ± 1.4	76.5 ± 2	70.2 ± 1.7	6.3
VO_2_@SiO_2_	48.9 ± 1.4	49.9 ± 1.4	55.0 ± 1.5	43.9 ± 1.4	11.1
VO_2_@TiO_2_	57.0 ± 1.2	53.7 ± 1.7	53.4 ± 1.4	43.0 ± 1.2	10.4

## Data Availability

The data presented in this study are available on request from the corresponding author.

## References

[1] Baetens R., Jelle B.R.P., Gustavsen A. (2010). Properties, requirements and possibilities of smart windows for dynamic daylight and solar energy control in buildings: A state-of-the-art review. Sol. Energy Mater. Sol. Cells.

[2] Wang S., Zhou Y., Jiang T., Yang R., Long Y. (2021). Thermochromic smart windows with highly regulated radiative cooling and solar transmission. Nano Energy.

[3] Lin C., Hur J., Chao C.Y.H., Liu G., Yao S., Li W., Huang B. (2022). All-weather thermochromic windows for synchronous solar and thermal radiation regulation. Sci. Adv..

[4] Lin Y., Zhe Q., Zili P., Jing L., Siyao T., Junhui H., Lili F. (2018). Three-Layered Hollow Nanospheres Based Coatings with Ultrahigh-Performance of Energy-Saving, Antireflection, and Self-Cleaning for Smart Windows. Small.

[5] Wang S., Jiang T., Meng Y., Yang R., Tan G., Long Y. (2021). Scalable thermochromic smart windows with passive radiative cooling regulation. Science.

[6] Gao Y., Luo H., Zhang Z., Kang L., Chen Z., Du J., Kanehira M., Cao C. (2012). Nanoceramic VO_2_ thermochromic smart glass: A review on progress in solution processing. Nano Energy.

[7] Yuanyuan C., Yujie K., Chang L., Zhang C., Ning W., Liangmiao Z., Yang Z., Shancheng W., Yanfeng G., Yi L. (2018). Thermochromic VO 2 for Energy-Efficient Smart Windows. Joule.

[8] Geng C., Zhang M., Wei H., Gu J., Zhao T., Guan H., Liang S., Boytsova O., Dou S., Chen Y. (2024). Tracking the optical constants of self-patterned VO_2_-based on smart windows during metal-insulator transition. Sol. Energy Mater. Sol. Cells.

[9] Hu L., Zhu H., Lu K., Wang C., Liu L., Ma L. (2024). Theoretical investigation of VO_2_ smart window with large-scale dynamic infrared emittance adjustment for adaptive thermal management. Sol. Energy.

[10] Zhu L., Tian L., Jiang S. (2023). Advances in photothermal regulation strategies:from efficient solar heating to daytime passive cooling. Chem. Soc. Rev..

[11] Ke Y., Chen J., Lin G., Wang S., Long Y. (2019). Smart Windows: Electro Thermo Mechano Photochromics, and Beyond. Adv. Energy Mater..

[12] Zhu M., Wang H., Li C., Qi H., Zhang D., Lv W. (2019). Thickness-modulated thermochromism of vanadium dioxide thin films grown by magnetron sputtering. Surf. Coat. Technol..

[13] Fan S., Wei X., Guo J., Guo R., Ren D., Zhao Z., Li J. (2025). Self-healing composite VO_2_ thin film with a thermochromic sandwich structure for smart windows. Ceram. Int..

[14] Jiang C., He L., Xuan Q., Liao Y., Dai J.-G., Lei D. (2024). Phase-change VO_2_-based thermochromic smart windows. Light Sci. Appl..

[15] Ji H., Zhao Y., Lu M., Tao J., Chen Y., Ou Y., Wang Y., Mao Y. (2023). Novel warm/cool-tone switchable VO_2_-based smart window composite films with excellent optical performance. Ceram. Int..

[16] Vu T.D., Xie H., Wang S., Hu J., Zeng X., Long Y. (2022). Durable vanadium dioxide with 33-year service life for smart windows applications. Mater. Today Energy.

[17] Qiao Y., Tang Z., Wu Z., Wang J., Sun X., Yu F., Wang C., Mao J., Zhang Q., Cao F. (2024). VO_2_-based colorful smart windows with self-cleaning function. Sol. Energy Mater. Sol. Cells.

[18] Kolenaty D., Houska J., Vlcek J. (2018). Improved performance of thermochromic VO_2_/SiO_2_ coatings prepared by low-temperature pulsed reactive magnetron sputtering: Prediction and experimental verification. J. Alloys Compd..

[19] Wang J., Li G., Zhao D. (2024). Multi-objective optimization of an anti-reflection AlN/VO_2_/AlN thermochromic window for building energy saving. Energy.

[20] Ou Y., Ji H., Wang Y., Liu B., Chen Y., Tao J., Huang Y., Wang J. (2024). Photothermal synergistic modulation of patterned VO_2_-Based composite films for smart windows. Sol. Energy.

[21] Ji Y., Mattsson A., Niklasson G.A., Granqvist C.G., Österlund L. (2019). Synergistic TiO_2_/VO_2_ Window Coating with Thermochromism, Enhanced Luminous Transmittance, and Photocatalytic Activity. Joule.

[22] Ma J., Ou W., Zhao L., Shi W., Zhu H., Liu J. (2025). Effect of SiO_2_ monocrystalline substrate orientation on the crystal structure and properties of VO_2_ film. J. Alloys Compd..

[23] Du Z., Li M., Zou F., Song Y., Xu S., Wu L., Li L., Li G. (2022). VO_2_@SiO_2_ Nanoparticle-Based Films with Localized Surface Plasmon Resonance for Smart Windows. ACS Appl. Nano Mater..

[24] Kang J., Liu J., Shi F., Dong Y., Song X., Wang Z., Tian Z., Xu J., Ma J., Zhao X. (2022). Facile fabrication of VO_2_/SiO_2_ aerogel composite films with excellent thermochromic properties for smart windows. Appl. Surf. Sci..

[25] Liang J., Li X., Zhang D., Wang S., Wang Z. (2022). Fabrication and optical performance research of VO_2_/SiO_2_/VO_2_ composite spherical structure films. J. Opt. Soc. Am. B.

[26] Zhong Q., Macharia K.D., Zhong W., Zixiao L., Zhigang C. (2020). Synthesis of hydrophobic W 18 O 49 nanorods for constructing UV/NIR-shielding and self-cleaning film. Ceram. Int..

[27] Jiang R., Cai W., Zheng T., Wang X., Yang F., Zhao H., Wang Y. (2025). Energy-Saving VO_2_ Bilayer Films with Self-Cleaning and Self-Healing for Smart Windows. ACS Appl. Mater. Interfaces.

[28] Alsaadi M., Lerttraikul K., Chatraphorn S., Kittiwatanakul S. (2024). Strategies for Enhanced Optical Performance and Durability of VO_2_-Based Thermochromic Windows. Adv. Eng. Mater..

[29] Zhao X.P., Mofid S.A., Gao T., Tan G., Jelle B.P., Yin X.B., Yang R.G. (2020). Durability-enhanced vanadium dioxide thermochromic film for smart windows. Mater. Today Phys..

[30] Powell M.J., Quesada-Cabrera R., Taylor A., Teixeira D., Papakonstantinou I., Palgrave R.G., Sankar G., Parkin I.P. (2016). Intelligent Multifunctional VO_2_/SiO_2_/TiO_2_ Coatings for Self-Cleaning, Energy-Saving Window Panels. Chem. Mater..

[31] Janiszewska E., Held A., Nowińska K., Kowalak S. (2019). One-pot synthesis of vanadium-containing silica SBA-3 materials and their catalytic activity for propene oxidation. RSC Adv..

[32] Vostakola M.F., Yekta B.E., Mirkazemi S.M. (2017). The Effects of Vanadium Pentoxide to Oxalic Acid Ratio and Different Atmospheres on the Formation of VO_2_ Nanopowders Synthesized via Sol–Gel Method. J. Electron. Mater..

